# Risk of Suicide and Self-Harm Following Gender-Affirmation Surgery

**DOI:** 10.7759/cureus.57472

**Published:** 2024-04-02

**Authors:** John J Straub, Krishna K Paul, Lauren G Bothwell, Sterling J Deshazo, Georgiy Golovko, Michael S Miller, Dietrich V Jehle

**Affiliations:** 1 Department of Emergency Medicine, University of Texas Medical Branch at Galveston, Galveston, USA; 2 Department of Pharmacology, University of Texas Medical Branch at Galveston, Galveston, USA; 3 Department of Psychiatry and Behavioral Services, University of Texas Medical Branch at Galveston, Galveston, USA

**Keywords:** self-harm, post traumatic stress disorder (ptsd), suicide, transgender, gender affirmation

## Abstract

Introduction

With the growing acceptance of transgender individuals, the number of gender affirmation surgeries has increased. Transgender individuals face elevated depression rates, leading to an increase in suicide ideation and attempts. This study evaluates the risk of suicide or self-harm associated with gender affirmation procedures.

Methods

This retrospective study utilized de-identified patient data from the TriNetX (TriNetX, LLC, Cambridge, MA) database, involving 56 United States healthcare organizations and over 90 million patients. The study involved four cohorts: cohort A, adults aged 18-60 who had gender-affirming surgery and an emergency visit (N = 1,501); cohort B, control group of adults with emergency visits but no gender-affirming surgery (N = 15,608,363); and cohort C, control group of adults with emergency visits, tubal ligation or vasectomy, but no gender-affirming surgery (N = 142,093). Propensity matching was applied to cohorts A and C. Data from February 4, 2003, to February 4, 2023, were analyzed to examine suicide attempts, death, self-harm, and post-traumatic stress disorder (PTSD) within five years of the index event. A secondary analysis involving a control group with pharyngitis, referred to as cohort D, was conducted to validate the results from cohort C.

Results

Individuals who underwent gender-affirming surgery had a 12.12-fold higher suicide attempt risk than those who did not (3.47% vs. 0.29%, RR 95% CI 9.20-15.96, p < 0.0001). Compared to the tubal ligation/vasectomy controls, the risk was 5.03-fold higher before propensity matching and remained significant at 4.71-fold after matching (3.50% vs. 0.74%, RR 95% CI 2.46-9.024, p < 0.0001) for the gender affirmation patients with similar results with the pharyngitis controls.

Conclusion

Patients who have undergone gender-affirming surgery are associated with a significantly elevated risk of suicide, highlighting the necessity for comprehensive post-procedure psychiatric support.

## Introduction

The prevalence of transgender individuals in the United States is approximately 0.3% to 0.6% of the adult population based on self-reporting studies [1]. Investigations that only include individuals with transgender diagnostic codes, hormone therapy, or gender-affirming surgery report a much lower rate of approximately 0.008% of the population [2]. People who identify as transgender are shown to have a higher risk of suicide in the United States and across many other countries [3-6].

In 2021, the Centers for Disease Control reported that 48,183 people died by suicide in the United States. Depression, substance abuse, other mental illness, legal/financial problems, harmful relationships, community risk factors, and easy access to lethal means are contributing factors to successful suicide. Transgender individuals have a higher prevalence of depression across several age groups, often due to life experiences that include discrimination, harassment, violence, misgendering, and enacted stigma that may generate poor mental health outcomes and harmful behaviors [4,7,8]. It is widely accepted that depression puts an individual at higher risk for suicidal ideation and suicide attempts [9]. Individuals at higher risk for suicide and post-traumatic stress disorder (PTSD) should have comprehensive psychiatric interventions and care throughout their lifetime. A better understanding of the relationship between suicide and gender affirmation remains particularly important.

There is ongoing controversy surrounding the benefits of gender-affirmation surgery on mental health [10-20]. This controversy reflects diverse perspectives within the medical and research communities, emphasizing the need for a more comprehensive understanding of the psychological outcomes of gender-affirming procedures. Despite the increasing acceptance of transgender individuals, questions persist about the psychological outcomes of gender-affirming procedures. Responses to the discussion surrounding the benefits of gender-affirmation surgery have been diverse, as evidenced by studies conducted by Branstrom and colleagues [11], Almazan et al. [13], and others [10,14-20].

The purpose of this study is to assess the risk of adverse outcomes, specifically suicide, death, self-harm, and PTSD in the five years following gender-affirmation surgery. Suicide risk over time among patients who received gender-affirmation surgery is compared to individuals in several control groups. The TriNetX (TriNetX, LLC, Cambridge, MA) database will be utilized to better understand the relationship between sex change and these outcomes.

This article was previously presented virtually as a meeting abstract at the 2023 Texas College of Emergency Physicians (TCEP) Research Forum on April 07, 2023.

## Materials and methods

TriNetX is a global health research network providing access to de-identified retrospective electronic medical records. The database consists of over 90 million patients from 56 healthcare organizations (HCOs) within the United States. This study utilized TriNetX to identify patients who had a “personal history of sex reassignment” and evaluate their relative risk for suicide attempt, death, suicide/self-harm, and PTSD. The term “sex reassignment” was based on the International Classification of Diseases, 10th Revision (ICD-10) code in the database but will be referred to as the current term “gender-affirmation surgery” for the remaining article. All outcomes were evaluated during the five years after gender-affirmation surgery.

Patients who have undergone gender-affirmation surgery of all sexes, races, and ethnicities were identified by using the ICD-10 code, ICD10CM:Z87.890. Sex, race, and ethnicity were derived from the electronic medical record. Patients who have undergone gender-affirmation surgery are identified by their affirmed gender. A total of four cohorts were identified for this study. Cohort A consisted of patients ages 18 to 60 who had both gender-affirmation surgery and an emergency visit. Cohort B was the study control group that consisted of patients ages 18 to 60 who had no history of gender-affirmation surgery but had an emergency visit. In this database, propensity matching is not possible for very large cohorts, more than 8.3 million patients with 12 covariates.

Additional control groups were chosen to perform propensity matching, which controls for confounders. Cohort C was the study's second control group and consisted of adult patients (18-60 years) who had no history of gender-affirmation surgery, had an emergency visit, and had a tubal ligation or vasectomy. Patients who had undergone tubal ligation were identified through the ICD-10 code, ICD10CM:Z98.51, while the vasectomy procedure was identified by Current Procedural Terminology (CPT), CPT:55250. A secondary sub-group analysis, cohort D, was performed utilizing acute pharyngitis (ICD10CM:J02) as a control group for patients aged 18-60 that was run on June 2, 2023. This was performed to ensure that the vasectomy or bilateral tubal ligation (BTL) group acted as an appropriate control. The relative risk for suicide attempt, death, suicide/self-harm, and PTSD was evaluated during the five years following gender-affirmation surgery in comparison to those without gender-affirmation surgery with the diagnosis of pharyngitis. Cohort A was used again and compared with cohort D, which included patients presenting to the emergency room after diagnosis of acute pharyngitis.

The outcome analysis between the three cohorts was performed for four events: suicide attempt (ICD10CM:T14.91), death (vital status: deceased), suicide/self-harm (ICD10CM:T14.91 or ICD10CM:X71-X83), and PTSD (ICD10CM:F43.1). An analysis was performed utilizing the measures contained in the TriNetX platform, which compared the individual outcomes between cohorts A and B and also cohorts A and C within the designated time frame. Patients who had the outcome before the time window were excluded from the analysis. The final TriNetX data reported RR, 95% CI, ORs, and a risk comparison expressed as a p-value. To control potentially confounding risk factors for the measured outcomes, the propensity matching tool in TriNetX was utilized. Factors involved in the data propensity matching are based on age at index, race, ethnicity, and sex. Propensity matching was only performed between the comparison of cohorts A and C, but not cohort B, due to the large sample size limitation.

Propensity score matching (PSM) is often used in observational studies to reduce confounding biases. It has been investigated and well-documented regarding its properties for statistical inference. PSM is a quasi-experimental method in which the researcher uses statistical techniques to construct an artificial control group by matching the affected group with a non-affected group of similar characteristics. Using these matches, the researcher can estimate the difference between both groups without the confounding variables’ influence [21]. To justify our use of propensity matching for age, race, sex, and ethnicity, we considered established risk factors for suicide such as older age, male gender identity, and racial or ethnic minority status [3,4].

The cohort was analyzed descriptively using univariate and bivariate frequencies with chi-square and t-testing to assess differences. All eligible persons in the cohort were analyzed using both binary event estimation with RRs, 95% CIs, and probability values. Using the TriNetX database, a 1:1 propensity match using linear and logistic regression for age, sex, race, and ethnicity was employed for maximum generalization of the United States population. Greedy nearest-neighbor matching was used with a tolerance of 0.1 and a difference between propensity scores less than or equal to 0.1. Comparisons were made between cohorts before and after propensity matching. Statistical significance was set at a two-sided alpha <0.05. TriNetX provides data that have been de-identified, and as a result, an Institutional Review Board (IRB) review is not required for this study [22]. Three comparison reports were generated on February 4, 2023. Data gathered from HCOs was from February 4, 2003, to February 4, 2023.

## Results

We identified 15,609,864 adult patients from TriNetX who were adults and had a visit to an emergency department within the United States Collaborative Network. Cohort A consisted of 1,501 adult patients who had a visit to the emergency department and a history of gender-affirmation surgery. Cohort B consisted of 15,608,363 patients who had an emergency visit but no history of gender-affirmation surgery. Cohort C consisted of 142,093 adult patients who had a visit to the emergency department and no history of gender-affirmation surgery but had a vasectomy or BTL.

Without propensity matching between cohorts A and B, patients with a history of gender-affirmation surgery exhibited a significantly higher risk for each possible outcome compared to patients without a history of gender-affirmation surgery (Table 1). Patients who had a history of gender-affirmation surgery had a 12.12 times greater risk of suicide attempts (3.47% vs. 0.29%, RR 95% CI 9.20-15.96, p < 0.0001) vs. patients who had no history of gender-affirmation surgery. In patients with a history of gender-affirmation surgery, there was a 3.35 times greater risk of being deceased (4.9% vs. 1.5%, RR 95% CI 2.673-4.194, p < 0.0001). Patients with a history of gender-affirmation surgery had a 9.88 times higher risk of self-harm or suicide (4.5% vs. 0.5%, RR 95% CI 7.746-12.603, p < 0.0001). Lastly, patients who had a history of gender-affirmation surgery had a 7.76 times higher risk of PTSD (9.2% vs. 1.2%, RR 95% CI 6.514-9.244, p < 0.0001).

**Table 1:** Outcomes following gender-affirmation surgery (cohort A) vs. no history of gender-affirmation surgery (cohort B) PTSD, post-traumatic stress disorder

Outcomes	Cohort A	Cohort B	RR (95% CI)	p-value
Suicide attempts	3.50%	0.30%	12.12 (9.202, 15.958)	<0.0001
Deceased	4.90%	1.50%	3.348 (2.673, 4.194)	<0.0001
Suicide or self-harm	4.50%	0.50%	9.880 (7.746, 12.603)	<0.0001
PTSD	9.20%	1.20%	7.760 (6.514, 9.244)	<0.0001

Before the propensity matching of cohorts A and C, there was a significantly higher risk for each outcome when considering patients with a history of gender-affirmation surgery compared to those without a history of gender-affirmation surgery but with a prior vasectomy or BTL (Table 2). Patients with a history of gender-affirmation surgery had a 5.03 times higher risk of suicide attempts (3.5% vs. 0.7%, RR 95% CI 3.795-6.676, p < 0.0001), a 2.37 times higher risk of being deceased (4.9% vs. 2.1%, RR 95% CI 1.889-2.982, p < 0.0001), a 5.44 times higher risk of suicide or self-harm (4.5% vs. 0.8%, RR 95% CI 4.233-6.981, p < 0.0001), and a 3.74 times higher risk of PTSD (9.2% vs. 2.5%, RR 95% CI 3.125-4.463, p < 0.0001) compared to patients without a history of gender-affirmation surgery but with a prior vasectomy or BTL.

**Table 2:** Outcomes following gender-affirmation surgery (cohort A) vs. vasectomy/tubal ligation (cohort C) before propensity matching PTSD, post-traumatic stress disorder

Outcomes	Cohort A	Cohort C	RR (95% CI)	p-value
Suicide attempts	3.50%	0.70%	5.03 (3.795, 6.676)	<0.0001
Deceased	4.90%	2.10%	2.37 (1.889, 2.982)	<0.0001
Suicide or self-harm	4.50%	0.80%	5.44 (4.233, 6.981)	<0.0001
PTSD	9.20%	2.50%	3.74 (3.125, 4.463)	<0.0001

After propensity matching of cohorts A and C, each cohort had 1,489 patients of similar age at index, race, and ethnicity (Tables 3-4). Patients who had a history of gender-affirmation surgery compared to patients without a gender-affirmation surgery history but had a vasectomy or BTL showed significantly higher risks for each outcome (Table 3). The adjusted suicide attempt risk for patients with gender-affirmation surgery compared to no history of gender-affirmation surgery but with a prior BTL or vasectomy was adjusted to a 4.71 times greater risk (3.50% vs. 0.74%, RR 95% CI 2.46-9.024, p < 0.0001). The risk of being deceased was 4.26 times greater in patients with a history of gender-affirmation surgery vs. patients with no history of gender-affirmation surgery but vasectomy or BTL (4.9% vs. 1.1%, RR 95% CI 2.520-7.191, p < 0.0001). Patients with a history of gender-affirmation surgery showed a 5.10 times higher risk of suicide or self-harm compared to patients with no history of gender-affirmation surgery but vasectomy or BTL (4.5% vs. 0.9%, RR 95% CI 2.816-9.227, p < 0.0001). Lastly, patients with a history of gender-affirmation surgery showed a 3.23 times higher risk for PTSD compared to patients with no history of gender-affirmation surgery but vasectomy or BTL (9.2% vs. 2.8%, RR 95% CI 2.278-4.580, p < 0.0001).

**Table 3:** Outcomes following gender-affirmation surgery (cohort A) vs. vasectomy/tubal ligation (cohort C) after propensity matching PTSD, post-traumatic stress disorder

Outcomes	Cohort A	Cohort C	RR (95% CI)	p-value
Suicide attempts	3.50%	0.74%	4.71 (2.46, 9.024)	<0.0001
Deceased	4.90%	1.10%	4.26 (2.520, 7.191)	<0.0001
Suicide or self-harm	4.50%	0.90%	5.10 (2.816, 9.227)	<0.0001
PTSD	9.20%	2.80%	3.23 (2.278, 4.580)	<0.0001

**Table 4:** Demographics before and after propensity score matching Cohort A: Adult patients who had a visit to the emergency department and a history of sexual reassignment. Cohort C: Adult patients who had a visit to the emergency department and no history of sexual reassignment but had a vasectomy or bilateral tubal ligation.

Demographics	Before propensity score matching		After propensity score matching	
	Cohort A (%)	Cohort C (%)	Cohort A (%)	Cohort C (%)
Total patients	1,501	142,093	1,489	1,489
Age at index ± SD	35.8 ± 11.6	41.1 ± 9.8	35.8 ± 11.6	36.3 ± 11.3
Female	760 (50.7%)	104,631 (76.1%)	760 (51.0%)	752 (50.5%)
Male	732 (48.8%)	32,830 (23.9%)	729 (49.0%)	737 (49.5%)
White	932 (62.1%)	90,431 (65.8%)	924 (62.1%)	892 (59.9%)
American Indian or Alaska Native	15 (1%)	483 (0.4%)	12 (0.8%)	32 (2.1%)
Native Hawaiian or other Pacific Islander	10 (0.7%)	147 (0.1%)	10 (0.7%)	10 (0.7%)
Hispanic or Latino	114 (7.6%)	15,780 (11.5%)	113 (7.6%)	111 (7.5%)
Black or African American	339 (22.6%)	27,253 (19.8%)	339 (22.8%)	345 (23.3%)
Asian	22 (1.5%)	2,073 (1.5%)	22 (1.5%)	19 (1.3%)
Not Hispanic or Latino	1,066 (71.1%)	87,544 (63.7%)	1,059 (71.1%)	1,060 (71.2%)
Unknown race	187 (12.5%)	17, 081 (12.4%)	187 (12.6%)	195 (13.1%)
Unknown ethnicity	320 (21.3%)	34,144 (24.8%)	317 (21.3%)	318 (21.4%)

The secondary sub-group analysis utilizing pharyngitis (N = 1,390,880) as a control revealed that patients presenting to the emergency department with a history of gender-affirmation surgery had a 7.95 times greater risk of suicide attempt than patients with pharyngitis (1.5% vs. 0.2%, RR CI 5.379-11.755, p < 0.0001), a 3.65 times greater risk of death (4.6% vs. 1.3%, RR CI 2.921-4.563, p < 0.0001), a 7.33 times greater risk of suicide or self-harm (2.7% vs. 0.4%, RR CI 5.448-9.850, p < 0.0001), and a 4.61 times greater risk of PTSD (9.2% vs. 2.0%, RR CI 3.901-5.438, p < 0.0001) compared to patients who were sent to the emergency department following acute pharyngitis. After propensity matching, mortality was 3.59 times greater in patients with a history of gender-affirmation surgery (4.6% vs. 1.3%, RR CI 2.224-5.806, p < 0.0001), and PTSD was 5.49 times greater (9.2% vs. 1.7%, RR CI 3.648-8.267, p < 0.0001) compared to patients with acute pharyngitis. There were too few suicides or self-harm outcomes to report results from the propensity-matched pharyngitis group. These results were similar to the results with cohort C.

## Discussion

The purpose of this study was to explore the relationship between gender-affirmation surgery and the risk of suicide outcomes compared to two control groups with data from 2003 to 2023. The significance of this investigation lies not only in its scale but also in its methodology, as it relies on real-world data rather than meta-analyses and self-reported surveys.

The first controlled group was a large number of patients who had emergency department visits but had not had gender-affirmation surgery. Propensity matching is not possible in the TriNetX database for large groups with millions of patients like the first control group. The second control group consisted of individuals who had not had gender-affirmation surgery but had either a vasectomy or BTL. This control group was selected to allow for propensity matching. Propensity matching was done for this comparison to control for the confounding influence of age, sex, and race/ethnicity. This is particularly important since the rate of successful suicide is much higher in men. At the start of this study, the hypothesis that was proposed predicted individuals who had undergone gender-affirmation surgery would have a greater risk of suicide, death, and self-harm compared to the two controls. This was confirmed by comparing the two control groups. In the second analysis, it was determined that patients who had undergone gender affirmation had a statistically significant increase in suicide attempts, death, self-harm, and PTSD after completion of gender affirmation in comparison with those who had undergone BTL or vasectomy and had not undergone gender-affirmation before propensity matching. After propensity matching our cohorts for age at index, race, and ethnicity, we also found a statistically significant increased risk of suicide attempts, death, self-harm/suicide, and PTSD. These outcomes confirmed the hypothesis. The secondary sub-group analysis utilizing pharyngitis as a control showed results that were comparable to the BTL/vasectomy control group, validating cohort C as an appropriate control group for propensity matching.

These data are supported by previous studies from multiple geographic regions of the globe, including Lebanon [3], Turkey [3], Pakistan [4], China [5], and Canada [6], as well as data from within the United States [3-4,6]. The large size of our study is an asset to our findings, which will help further our understanding of the relationship between sex change and suicide. To our knowledge, a study of this size has not been described in the literature. Using two control groups, a) those who had not experienced gender-affirmation surgery and had presented to the emergency department and b) a group that had not experienced gender-affirmation surgery, had visited the emergency department, and had a vasectomy or BTL, also helped effectively control for confounding variables utilizing propensity matching. Over the last 20 years, this study demonstrated a 12.12 times greater risk of suicide utilizing the first control group and a 4.71 to 5.03 times increased risk with the other control groups.

Transgender individuals, encompassing both those seeking gender-affirming surgery and those who have undergone it, demonstrate a significantly elevated risk of developing PTSD compared to the general population [10,23]. Among those who seek access to gender-affirming surgery, the commonality of discrimination, interpersonal assault, and a lack of social support have been identified as influential factors in the development of PTSD within this group [23]. Financial stress and insufficient insurance coverage prove to be significant obstacles for those trying to access gender-affirming surgery. Additionally, the limited availability of medical professionals with expertise in gender-affirming procedures, particularly in areas of lower socioeconomic status, further exacerbates the challenges faced by individuals seeking such care [10]. However, it is important to consider PTSD development in those who have undergone gender-affirming procedures. The emergence of PTSD following surgery often stems from the pre-operative challenges (such as harassment, limited social support, etc.) in conjunction with suboptimal surgical outcomes and insufficient psychiatric assistance.

This study has revealed a significantly elevated prevalence of PTSD in post-operative transgender individuals, with a 7.76-fold increase in comparison to cohort B and a 3.74-fold increased risk compared to cohort C after propensity matching. These findings were consistent with other studies investigated previously. A study conducted by Livingston et al. in 2022 used probabilistic and rule-based modeling on Veterans Health Administration (VHA) records from 1999 to 2021 to assess the differences in PTSD prevalence among 9,995 transgender and 29,985 cisgender veterans (1:3 ratio). They concluded that transgender veterans experienced PTSD at 1.5-1.8 times the rate of veterans identifying as cisgender, especially higher in recent users of VHA services [24]. There have proven to be many obstacles when comparing our findings to other studies assessing general population PTSD risk in those who have undergone gender-affirmation surgery. A 2018 systematic review conducted by Valentine et al. showed that many studies used assessment tools not particularly appropriate for evaluating mental health in transgender or gender non-conforming individuals [25]. The poor psychometric framework has led to many studies not acknowledging confounding and contextual variables, such as exposure to discrimination or minority identity when assessing PTSD in this demographic [10,25]. To avoid the repeated shortcomings of prior research, future studies should employ rigorous and reliable assessment tools such as cross-sectional studies or the collection of prospective data [25]. Improving transgender representation in emerging PTSD treatment trials is another step in improving the understanding and management of PTSD in transgender individuals [10].

In light of the examination of the relationship between gender-affirmation surgery and mental health outcomes discussed in this study, it is imperative to acknowledge the broader landscape of research on this topic. Our investigation contributes broad insight, examining real-world data over two decades and encompassing a diverse cohort. However, to further expand upon the contextual significance, it is essential to compare findings from other studies that explore multifaceted aspects of mental health post-gender-affirmation surgery. A study published in the American Journal of Psychiatry by Branstrom et al. in October 2019 drew strong conclusions regarding the positive impact of gender-affirmation surgery on mental health [11]. However, the study faced criticism of its methodology, leading to a correction/retraction by the journal's editors that stated, "the results demonstrated no advantage of surgery about subsequent mood or anxiety disorder-related health care visits or prescriptions or hospitalizations following suicide attempts” [12]. In a subsequent study conducted in 2021, Almazan et al. compared the mental health outcomes of a group of patients who were not approved for gender-affirmation surgery with a group that had undergone the surgery [13].

Their findings suggested better mental health outcomes for those who underwent surgery, but notable limitations warrant careful interpretation. First, the study conducted a comparison between two groups: one that had not been approved for surgery, a process requiring two mental health screenings as per the World Professional Association for Transgender Health's standard of care recommendations, and another group that had already undergone surgery. Therefore, it is plausible that the surgery group could inherently have been healthier, irrespective of the surgery. Second, when the analysis was broadened to include lifetime outcomes, the positive association with the surgery became insignificant [14].

Although our study has revealed a statistically significant increase in suicide risk among those who have undergone gender-affirming surgery, it remains vital to recognize and support the positive impacts that these surgical interventions can have on the lives of transgender individuals. The results of a study by Park et al., published in October 2022 in the Annals of Plastic Surgery, provide a different perspective on the enduring effectiveness and consequences of gender-affirmation surgery [20]. While our research specifically examined the risk of suicide, death, self-harm, and PTSD in the five years following surgery, Park et al. surveyed the outcomes of 15 gender-affirming surgeries over a more extended period. Their results reveal an improvement in patient well-being, with high satisfaction levels, reduced dysphoria, and persistent mental health benefits even decades after surgery. Notably, the study highlights the durability of these positive outcomes and significantly reduced suicidal ideation following gender-affirmation surgery.

The number of non-gender-conforming individuals continues to increase globally. It is likely, therefore, that a growing number of medical professionals will care for an individual who has undergone gender-affirmation at some point in their career. Apart from additional assistance in surgical recovery, the most common aftercare needs for patients following gender-affirmation surgery is consultation with a mental health professional [26]. To properly address the mental health needs of transgender individuals, Lapinski et al. emphasize the significance of cultural competency, a patient-focused approach, and collaborative efforts involving psychiatric professionals [27-30]. Transgender individuals tend to see mental health care providers and face discrimination in clinical settings at a far higher rate than the cis-gendered population [27,28,30]. Competent medical care following gender-affirming surgery is vital in effectively managing PTSD and its respective mental health challenges for this population [27].

It is important to note that this study has several limitations. The retrospective cohort design can only demonstrate associations but not causality. However, the larger size of this study, in conjunction with propensity matching, gives this investigation a greater power to identify differences between groups. Additionally, with the extensive timeline of data collection, the findings are relevant and contemporary to modern situations. A limitation of the study design could include the fact that only adult data was analyzed, so the research cannot be generalized to those under the age of 18. The data were also only extracted from a population of residents from the United States. Patients who have undergone gender-affirmation surgery and our control groups may have refrained from disclosing their suicidal ideations or other psychiatric symptoms to their medical providers, potentially influenced by societal pressures or other factors such as perceived attitudes toward those with psychiatric complaints. It may be worth examining if groups considering gender-affirmation surgery who have not yet received the surgery share the same increased risk levels for suicidal actions and ideations. However, given the standard practice of undergoing psychiatric testing before being approved for gender-affirmation surgery, individuals contemplating the procedure may potentially pose a greater suicide risk compared to those who have been approved for surgery.

## Conclusions

The results of this study indicate that patients who have undergone gender affirmation surgery are associated with significantly higher risks of suicide, self-harm, and PTSD compared to general population control groups in this real-world database. With suicide being one of the most common causes of death for adolescent and middle-aged individuals, it is clear that we must work to prevent these unfortunate outcomes. This further reinforces the need for comprehensive psychiatric care in the years that follow gender-affirmation surgery.

## Appendices

Greedy nearest-neighbor matching

The most common implementation of propensity matching is pair-matching, in which pairs of treated and control participants are formed. There are several common implementations of pair-matching. The most commonly used is greedy nearest-neighbor matching (NNM), which we used, in which a treated participant is selected at random and then matched to the control participant whose propensity score is closest to that of the treated participant. The process is described as greedy because, at each stage, the control is selected who is closest to the currently considered treated participant, even if that untreated participant would serve better as a control for a subsequently treated participant. This process is then repeated until a matched control participant has been selected for each treated participant. This process generally uses matching without replacement so that once a control participant is matched to a treated participant, that control participant is no longer available to match to a subsequently treated participant. A reﬁnement to NNM is NNM with a caliper restriction. Using this approach, a control participant is an acceptable match for a treated participant only if the diﬀerence in their propensity scores is less than a maximum amount (the caliper width or distance). For technical reasons, one typically matches the logit of the propensity score and uses a caliper width that is deﬁned as a proportion of the (0.1-0.2) SD of the logit of the propensity score. A crucial step in any study that uses PSM is to assess the degree to which matching the propensity score resulted in the formation of a matched sample in which the distribution of baseline characteristics is similar between treated and control participants. This assessment is critical as it allows both the researcher and readers to assess whether matching the estimated propensity score has removed systematic baseline diﬀerences between treatments. The use of the standardized diﬀerence, which is the diﬀerence in means in units of SD, is often used for assessing the similarity of matched treated and control participants. Some authors have suggested that a threshold of 0.10 (or 10%) be used to denote acceptable balance after matching. Once an acceptable balance has been achieved, analysts can unblind themselves to the outcome and compare outcomes between treated and control participants in the matched sample. The analyses conducted in the propensity score-matched sample can be similar to those that would be done in an RCT with a similar outcome.
