# Correction: Risk of Suicide and Self-Harm Following Gender-Affirmation Surgery

**DOI:** 10.7759/cureus.c182

**Published:** 2024-06-11

**Authors:** John J Straub, Krishna K Paul, Lauren G Bothwell, Sterling J Deshazo, Georgiy Golovko, Michael S Miller, Dietrich V Jehle

**Affiliations:** 1 Department of Emergency Medicine, University of Texas Medical Branch at Galveston, Galveston, USA; 2 Department of Pharmacology, University of Texas Medical Branch at Galveston, Galveston, USA; 3 Department of Psychiatry and Behavioral Services, University of Texas Medical Branch at Galveston, Galveston, USA

This article has been corrected by the Editors-in-Chief to clarify the conclusions of the study. Two sentences have been revised as detailed below. The authors agree with these corrections.

- Original abstract conclusions: "Gender-affirming surgery is significantly associated with elevated suicide attempt risks, underlining the necessity for comprehensive post-procedure psychiatric support." Corrected to: "Patients who have undergone gender-affirming surgery are associated with a significantly elevated risk of suicide, highlighting the necessity for comprehensive post-procedure psychiatric support."
 
- Original first sentence of the Conclusions section: "The results of this study show that gender-affirmation surgery is associated with a significantly higher risk of suicide, death, suicide/self-harm, and PTSD compared to control groups in this real-world database." Corrected to: "The results of this study indicate that patients who have undergone gender affirmation surgery are associated with significantly higher risks of suicide, self-harm, and PTSD compared to general population control groups in this real-world database."

